# Combined use of a microbial restoration substrate and avirulent *Ralstonia solanacearum* for the control of tomato bacterial wilt

**DOI:** 10.1038/s41598-019-56572-y

**Published:** 2019-12-27

**Authors:** Xuefang Zheng, Yujing Zhu, Jieping Wang, Ziran Wang, Bo Liu

**Affiliations:** 1 Agrobiological Resource Research Institute, Fujian Academy of Agriculture Sciences, Fuzhou, 350003 China; 2 Dean of Department of Biochemistry and Biotechnology, School of Life Sciences, Xiamen University, Xiamen, 361102 China

**Keywords:** Applied microbiology, Plant development

## Abstract

Tomato bacterial wilt (BW) caused by *Ralstonia solanacearum* seriously restricts tomato production and no effective control measures are available. A microbial restoration substrate (MRS) had been proved to be effective control of tomato BW in a greenhouse cultivation. In this study, MRS was combined with an avirulent *Ralstonia solanacearum* (aRS) strain to control the disease under an open field condition. In the two consecutive year (2017 and 2018) trials, the combined use of aRS and MRS resulted in better disease control compared with either aRS or MRS alone. Moreover, the combined treatment was more effective than expected and suggesting a synergistic control effect. Compared with control (CK, non-aRS or MRS), the application of aRS and MRS treatments alone or in combination could all promote plant growth, increase root activity and yield (e.g. the yield for the treatment of aRS + MRS increased by 463.64% in 2017). Soil nutrients, including soil organic carbon, total nitrogen, total phosphorus and total potassium contents were also significantly increased by the application of aRS and MRS treatments alone or in combination (P < 0.05). The application of MRS or in combination with aRS changed the soil from acidic to neutral, which is one of the key factors for controlling BW. The soil enzymatic activities were notably influenced by the combined use of aRS and MRS, which increased urease (87.37% in 2017 and 60.89% in 2018), catalase (93.67% in 2017 and 279.37% in 2018) and alkaline phosphatase activities (193.77% in 2017 and 455.73% in 2018). These results suggest that the combination of MRS and aRS could effectively control tomato BW and thus represents a promising new tool to control this disease.

## Introduction

The tomato (*Solanum Lycopersicum* L.) is one of the world’s main vegetable crops, and it is cultivated worldwide for fresh vegetable consumption or for processing^1^. A large portion of tomato crop has been grown by continuous cropping for many years in the world. This practice has led to the outbreak of the destructive soilborne disease ‘bacterial wilt (BW, caused by virulent *Ralstonia solanacearum*)’, which can eventually causes a complete yield loss^2^. Many measures have been studied to control the disease, either by inhibiting pathogen growth within the rhizosphere or by inducing host plant resistance^3–6^, but limited success has been achieved due to the high surviving capacity of *R*. *solanacearum* in complex environments^7^.

Currently, biocontrol emerges as an environmentally friendly strategy and popular method to suppress tomato BW disease^8,9^, and several biocontrol agents have been isolated from the rhizosphere soil or plant tissues, such as *Bacillus* spp.^10^, *Streptomyces* spp.^11^ and avirulent mutants of *R*. *solanacearum*^12–14^, which have shown antagonistic effects against pathogenic *R*. *solanacearum*. Many literatures reported that the avirulent mutants of *R*. *solanacearum* can effectively reduce bacterial wilt severity^15–17^. A high density of avirulent *R*. *solanacearum* within plant tissues might influence host’s response to pathogen and prevent its wilting^15^. In our previous studies, the avirulent *R*. *solanacearum* strain FJAT-1458 was isolated from a healthy tomato plant with high purity and proved to have high biocontrol efficiency against BW of 100 and 77.45% under pot and greenhouse cultivations, respectively^18,19^.

Successful control of soilborne diseases by soil amendments is well documented^5,10,20,21^. Mathre *et al*. (1999) reported that soil remediation with organic matter brought soil-sanitization effects and then suppressed soilborne diseases^20^. Ding *et al*.^5^ and Yuan *et al*.^10^ combined the use of organic fertilizers and bacterial antagonists to control potato (*Solanum tuberosum* L.) and tobacco (*Nicotiana tabacum* L.) BW, respectively, and successfully decreased bacterial disease incidences. In our previous study, a type of microbial restoration substrate (MRS) was used to amend 7-year continuous cropping tomato soil under a greenhouse cultivation and proved to be effective in reducing the severity of BW^22^. Soil pH, rhizobacterial community and soil nutrient composition were all changed by MRS application.

Due to variable environmental conditions and the complexity of plant and soil systems, it is difficult to achieve satisfactory disease control using a single control method^23^. Guo *et al*.^24^ found that the suppression efficacy of antagonist inoculation against BW was inconsistent across field trials and was influenced by environmental conditions. Wu *et al*.^25^ reported that the application of biocontrol agents with organic fertilizers was more effective in controlling tobacco BW than the sole application of biocontrol agents. Peng *et al*.^26^ combined the use of *Bacillus subtilis* and bactericide Saisentong (N,N-methylene-bis-(2-amino-5-sulfhydryl-1,3,4-thiadiazole) copper) to control tomato BW, and this resulted in better disease control compared with either agent alone. Therefore, integrating different methods to manage tomato BW should be a feasible approach under field conditions.

Based on previous studies under greenhouse cultivation, we further investigated a combination of the biocontrol agent *R*. *solanacearum* FJAT-1458 with the soil amendment MRS in control of tomato BW under open field cultivation. The objectives of this study were (i) to evaluate the potential biocontrol abilities of the avirulent strain FJAT-1458 in combination with the microbial restoration substrate against BW, and (ii) to investigate their mode of action and effect on properties of tomato rhizosphere soils.

## Materials and Methods

### Bacterial culture

The avirulent strain of *R*. *solanacearum* FJAT-1458 used in this study was grown at 30 °C for 48 h on 2,3,5-triphenyltetrazolium chloride (TTC) medium (1% peptone, 0.5% glucose, 0.1% trypticas, 1.8% agar, and 0.05% 2,3,5-triphenyltetrazolium chloride)^27^. Then, a single colony was suspended in liquid sucrose peptone (SP) medium, which contained 20 g·L^−1^ sucrose, 5 g·L^−1^ peptone, 0.5 g·L^−1^ K_2_HPO_4_, and 0.25 g·L^−1^ MgSO_4_, at a pH between 7.2 and 7.4, and cultured in a shaker at 30 °C, 200 rpm for 48 h. The concentration of the cultured bacteria reached to 10^9^ CFU mL^−1^ and bacterial cells density was diluted to 10^7^ CFU mL^−1^ by water, when being used in the field.

### Field experiments

The field experiment was conducted for two consecutive years, from August to December, in 2017 and 2018 at Licheng district, Putian City, Fujian Province, in southeastern of China (25°45′N, 118°32′E). Putian City is located in a subtropical monsoon climate (it has an annual rainfall of 1,500 mm, and the annual average temperature is 20 °C). A tomato field undergoing 15 years of continuous tomato cropping, which exhibited severe BW in previous years (disease incidence was over 50%), was selected for the experiment. The tomato (cv. Beiying) seeds were sown in 32-hole plugs on August 20 and were grown in a greenhouse at 25–30 °C at 85–100% relative humidity. The seedlings were transplanted into an open field after 1 month. Prior to the tomato planting, 160 kg hm^−2^ of nitogen (N) fertilizer,120 kg hm^−2^ of phosphate (P) fertilizer and 320 kg hm^−2^ of potassium (K) fertilizer were applied as base fertizer. Then, 100 kg ha^−1^ of K fertilizer was applied at fruit setting.

**Figure 1:**
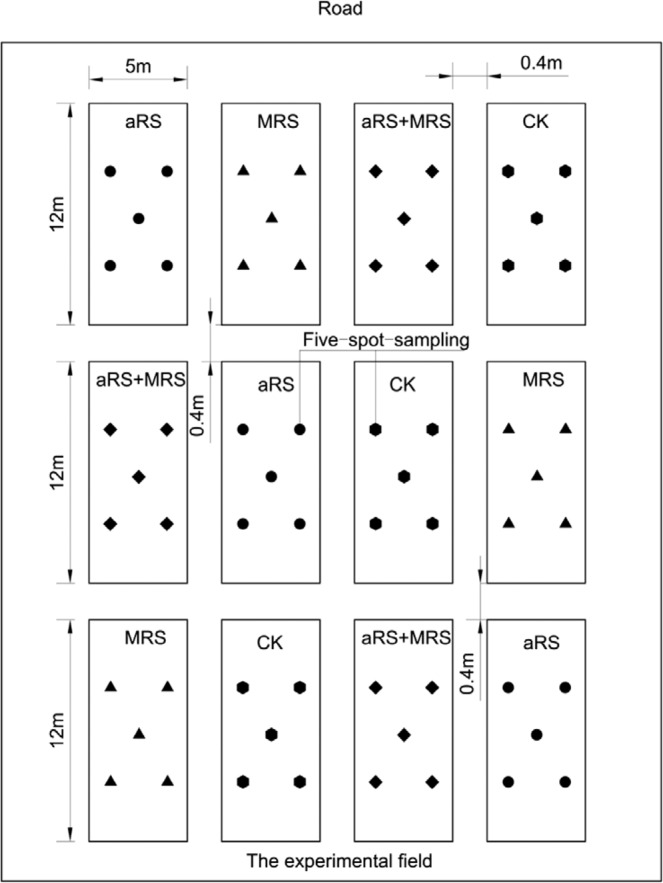
Diagram of different treatment plots in the field. aRS, avirulent *Ralstonia solanacearum* strain FJAT-1458 inoculation alone; MRS, soil amendment with microbial restoration substrate alone; aRS + MRS, combination of aRS and MRS; CK, non-(aRS or MRS) was used as control. Five solid circles in each plot of aRS, five solid triangles in each plot of MRS, five solid rhombus in each plot of aRS + MRS, five solid hexagon in each plot of CK represent of five-spot-sampling of rhizosphere soils for aRS, MRS, aRS + MRS and CK, respectively.

Four treatments were established as follows: (1) aRS, which was the avirulent strain FJAT-1458 inoculation alone; (2) MRS, which was soil amendment with microbial restoration substrate alone; (3) aRS + MRS, which was the combination of aRS and MRS; and (4) CK, in which neither soil amendment nor avirulent strain inoculation were used. Randomized block design and triplicate plots were created for the experiment. Each plot consisted of five 12 m-long rows, corresponding to a total 60 m^2^ plot area, and 216 plants per plot. The distance between adjacent plots was 0.4 m (Fig. 1). For the soil amendment, 1,500 kg ha^−1^ microbial restoration substrate was furrow applied two days (d) before tomato plant transplanting. For the avirulent strain FJAT-1458 inoculation, the plants were root drenching inoculated with FJAT-1458 (10^7^ CFU mL^−1^, 500 mL per plant) at transplanting, and the root drenching inoculation was repeated one month later. The MRS was jointly produced by the Fujian Academy of Agriculture Sciences (FAAS) and Xiamen Jiang Ping Biological Co., Ltd., China (XJPBC), using a microbial fermentation bed^28^. The pig manure nitrogen was continuously added to achieve aerobic fermentation in medium temperature. The fully manufacturing processes of MRS were as follows: the litters consisting of 33% chaff, 33% coir and 34% rice straw were added onto the pig microbial fermentation bed; the aerobic fermentation was conducted for 20 days by ploughing the litters mixing with pig manures one time per day; then, the upper 20 cm litters were removed to produce MRS by drying, crushing, screening and packaging. Illumina-MiSeq sequencing of the MRS, showed that the dominant bacterial genera were *Granulicella* (3.31%), *Acidothermus* (3.20%) and *Rhodanobacter* (1.27%) (NCBI accession number: SRP144025). The physiochemical characteristics of MRS were as follows: pH 7.82, the soil organic carbon (SOC) 145.12 g kg^−1^, total nitrogen (TN) 4.62 g kg^−1^, total phosphorus (TP) 3.18 g kg^−1^, total potassium (TK) 16.51 g kg^−1^, and exchangeable calcium 28.54 g kg^−1^.

### Soil sampling and disease investigation

Rhizosphere soil samples (attached to the root surface) were collected at the reproductive stage of tomato plants (90 d after transplanting). Each soil sample was taken from 25 plants in each block by the five-spot-sampling method (Fig. 1) and was partitioned into three subsamples, one for the enzyme assay, one for the biochemical property tests, and one for *R*. *solanacearum* detection.

The severity of tomato BW was investigated at the reproductive stage (90 d after transplanting). Based on the observations of leaf wilt symptoms, tomato bacterial wilt severity was empirically categorized into five grades as follows: 0, no wilting; 1, 1% to 25% wilting; 2, 26% to 50% wilting; 3, 51% to 75% wilting; and 4, 76% to 100% wilting or death of the entire plant^29^. The disease incidence, control efficiency, and disease severity index (DSI) were calculated as follows:

Disease incidence = ∑ (number of diseased plants/total number of plants investigated)

Control efficiency = [(disease incidence of CK - disease incidence of treatment aRS or MRS or “aRS + MRS”)/disease incidence of CK] × 100%

DSI = (4 A + 3B + 2 C + 1D)/N × 100, where A represents the number of plants in grade 4, B represents the number of plants in grade 3, C represents the number of plants in grade 2, D represents the number of plants in grade 1, and N represents the total number of plants^30^.

### *Ralstonia solanacearum* detection

**Figure 2:**
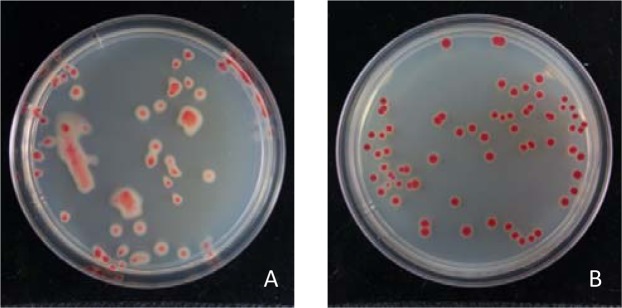
Colony morphology of virulent (**A**) and avirulent (**B**) *Ralstonia solanacearum* strains on TTC medium. The isolated *R*. *solanacearum* from the test samples showed two different colony morphologies on TTC medium. The colony of virulent *R*. *solanacearum* was irregular, highly mobile, humid and displayed a pink spot in the middle of the colony and a large white edge (**A**); while the colony of avirulent *R*. *solanacearum* was round, immobile, dry and displayed a dark red spot in the middle of colony and a narrow or no white edge (**B**).

The quantification of *R*. *solanacearum* inoculum in the rhizosphere soil of tomato plants was determined by adding 10 g soil to 90 mL distilled water and shaking thoroughly. The suspension was subjected to a serial dilution, placed onto TTC medium to isolate *R*. *solanacearum*, and cultured at 30 °C for 48 h. The isolated *R*. *solanacearum* strains were confirmed by PCR with primers pehA#6/pehA#3 according to Gillings *et al*.^31^. Based on the colony morphology on TTC medium, the viable colonies were counted for each sample. According to Kelman^27^ and Liu *et al*.^32^, the colony of a virulent *R*. *solanacearum* strain is irregular, highly mobile, humid and displays a pink spot in the middle of the colony and a large white edge (Fig. 2A), while the colony of an avirulent *R*. *solanacearum* strain is round, immobile, dry and displays a dark red spot in the middle of colony and a narrow or no white edge (Fig. 2B).

### Biological investigations

At vegetative stage (30 d after transplanting), some biological characteristics were investigated, including plant height, stem diameter (basal part of stem), and root activity. At harvest stage, the yield was also measured. For each treatment, 30 plants were selected using five-spot-sampling method to measure the plant height and stem diameter. The tomato roots of different treatments were collected (10 g) and their activity was measured using 2,3,5-triphenyl tetrazolium chloride (TTC) reduction method^33^. The tomato fruits of each treatment were weighed separately at per harvest time and the yields were calculated.

### Enzyme assay

The soil samples were air-dried at room temperature for 48 h and passed through a 2 mm sieve for the enzyme activity analyses. All enzyme activities were assayed in triplicate with one control. The activities of urease, catalase, alkaline phosphatase and sucrase that are generally used to evaluate fertility and quality of soil were determined according to Guan^34^ with some minor modifications.

For urease activity assay, 5 g of soil was mixed with 5 mL of toluene, 10 mL of 10% urea solution, and 20 mL of distilled water, shaken and incubated at 37 °C for 24 h. Then, the soil suspension was centrifuged at 8,000 rpm for 5 min. A 0.5 mL of supernatant was treated with 4 mL of a mixed reagent (100 mL of a 6.6 M phenol solution and 100 mL of 6.8 M NaOH) and 3 mL of a sodium hypochlorite solution. The volume was adjusted to 20 mL with distilled water. The urease activity was quantified colorimetrically at 578 nm under UV-visible spectrophotometer (UV-2550, Shimadzu, Japan) and was expressed as μg NH_4_-N ·g^−1^ soil·  24 h^−1^.

For catalase activity assay, 3 g of soil was mixed with 5 mL of 0.3% H_2_O_2_ and 40 mL distilled water. The soil slurry was shaken thoroughly for 20 min. Subsequently, 5 mL of 3 N H_2_SO_4_ was added to remain peroxide stability and the suspension was then centrifuged at 8,000 rpm for 5 min. The catalase activity was determined by titration with 0.1 M KMnO_4_ and expressed as mL KMnO_4_·g^−1^ soil·20 min^−1^.

For alkaline phosphatase activity assay, 2 g of soil was mixed with 2 mL of toluene and 20 mL of borate saline buffer (pH 9.6). The soil slurry was incubated at 37 °C for 24 h. Then 40 mL of a 3% aluminum sulfate solution was added, and it was centrifuged at 8,000 rpm for 5 min. After centrifugation, 3 mL of supernatant was mixed with four drops of 2,6-dibromoquinone-4-chloroimide and was then diluted to 50 mL. The alkaline phosphatase activity was quantified colorimetrically at 660 nm under UV-visible spectrophotometer (UV-2550, Shimadzu, Japan) and was expressed as *μ*g phenol·g^−1^ soil·24 h^−1^.

For sucrase activity assay, 5 g of soil was mixed with 5 mL of toluene, 15 mL of an 8% sucrose solution and 5 mL of phosphate buffer (pH 5.5). Then the soil slurry was inoculated at 37 °C for 24 h and was then centrifuged at 8000 rpm for 5 min. The suspension (1 mL) was added with 3 mL of salicylic acid and was incubated at 100 °C for 5 min, cooled for 3 min with flowing tap water and was then added with distilled water to make up 50 mL. The sucrase activity was measured under UV-visible spectrophotometer (UV-2550, Shimadzu, Japan) at an absorbance of 508 nm and was expressed as mg ·glucose ·g^−1^ soil·24 h^−1^.

### Physicochemical assay

The soil samples were air-dried at room temperature and passed through a 2 mm sieve for physicochemical analyses. The soil organic carbon (SOC) was measured by the Walkley-Black method^35^, the total nitrogen (TN) was determined by the Kjeldahl method^36^ the total potassium (TK) was determined using an atomic absorption spectrophotometer after wet digestion of soil sample with NaOH^37^, the total phosphorus (TP) was determined by Vanado-Molybdate phosphoric yellow colorimetric procedure and the exchangeable calcium was determined using atomic absorption spectrophotometry after extracting with ammonium acetate^38^. The soil pH was measured using a 1:2.5 (w:v) soil: water ratio.

### Data analysis

All statistical analyses were performed using the SPSS 13.0 software (SPSS Inc, Chicago, IL). Differences of the control efficiency, *R*. *solanacearum* population, soil physicochemical property and enzymatic activity among the treatments and years were assessed with two-way analysis of variance (ANOVA). The mean comparisons were made using ANOVA and a least significant difference (LSD) test (*P* < 0.05). The correlation analysis between the disease incidence and the soil chemical properties was conducted by a Pearson’s correlation. The synergistic responses of the MRS and aRS in the control efficiency were calculated using the method introduced by Colby^39^: *E* = *I*_f_ + *I*_b_ − *I*_f_ *I*_b_/100, where *I*_f_ is the observed control efficiency of MRS, *I*_b_ is the observed control efficiency of aRS, and E is the expected control efficiency of the combination MRS and aRS. When the observed response was greater than expected, the combination was considered synergistic, and vice versa.

## Results

### Control of tomato BW by different treatments

The effect of MRS combined with aRS on tomato BW control was evaluated in a field experiment. The disease incidence of the aRS + MRS treatment was the lowest among the four treatments for the two consecutive years (Table 1). The combined use of aRS and MRS yielded better control of tomato bacterial wilt. The control efficiency of treatment aRS + MRS was 80.79% in 2017 and 85.79% in 2018, which was significantly higher than aRS (63.06% in 2017 and 63.79% in 2018) (p-value = 0.0001) and MRS (42.86% in 2017 and 52.64% in 2018) (p-value = 0.0001). Moreover, the observed control efficiency of the combined use of aRS and MRS was higher than the expected control efficiency, suggesting a synergistic control effect. Two-way ANOVA showed that the difference of control efficiency among different treatments reaching to significant level, but it was not for different years (except for MRS, p-value = 0.0021).

**Table 1:** Control of tomato bacterial wilt by an avirulent *Ralstonia solanacearum* strain and a microbial restoration substrate.

Treatment	2017				2018			
	Disease severiy index	Observed control (%)	Expected control (%)	Difference	Disease severiy index	Observed control (%)	Expected control (%)	Difference
CK	43.18 ± 2.12 a	—	—	—	53.10 ± 4.18 a	—	—	—
aRS	14.49 ± 0.67c	63.06 ± 0.91 b	—	—	14.52 ± 1.58 b	63.79 ± 1.14 c	—	—
MRS	19.24 ± 1.91 b	42.86 ± 1.18 c	—	—	16.32 ± 1.65 b	52.64 ± 1.99 b	—	—
aRS + MRS	8.59 ± 1.06 d	80.79 ± 3.87 a	78.89 ± 0.08	+1.90	6.13 ± 0.87 c	85.79 ± 1.83 a	82.86 ± 0.92	+2.93

Data are means ± standard error (n = 3); Values within a row followed by the same letter are not significantly different at P ≤ 0.05.

^a^Reduction in disease incidence relative to the control.

^b^Expected control is the cotrol resulting from the combination as predicted by the equation of Colby.

^d^Difference = % reduction observed-% reduction expected, with a plus sign indicating a synergistic

effect.

### Quantification of *R. solanacearum* in the tomato rhizosphere soils after the different treatments

Compared to CK, the application of aRS, MRS and aRS + MRS treatments significantly reduced the population of virulent *R*. *solanacearum* by 96.64, 81.56 and 99.72% in 2017 (p-value = 0.0001), and by 97.62, 98.95 and 99.77% in 2018 (p-value = 0.0001) (Table 2). However, The avirulent *R*. *solanacearum* was detected only in the treatments of aRS and aRS + MRS. The population of avirulent *R*. *solanacearum* in the treatment of aRS + MRS was significantly higher than in the treatment of aRS (p-value = 0.0007).

**Table 2:** Quantification of *Ralstonia solanacearum* in the tomato rhizosphere soil (×10^5^ cfu g^−1^).

Treatments	Experiment time (year)	Avirulent strain (×10^5^ cfu g^−1^)	Virulent strain (×10^5^ cfu g^−1^)
CK	2017	0 d	53.62 ± 7.51 b
aRS		0.59 ± 0.03 c	1.80 ± 0.21 d
MRS		0 d	9.89 ± 0.94 c
aRS + MRS		2.57 ± 0.07 b	0.15 ± 0.04 d
CK	2018	0 d	60.00 ± 6.82 a
aRS		0.53 ± 0.12 c	1.43 ± 0.17 d
MRS		0 d	0.63 ± 0.11 d
aRS + MRS		3.07 ± 0.45 a	0.14 ± 0.01 d

Data are means ± standard error (n = 3); Values within a row followed by the same letter are not significantly different at P ≤ 0.05.

### Biological characteristics of tomato plants after the different treatments

The application of MRS, aRS alone or in combination could all increase plant growth, root activity and yield in comparison to CK (Table 3). Plants of treatment aRS + MRS had greater plant heights than those subjected to the other treatments. The stem diameters for treatments aRS + MRS and MRS were significantly greater than aRS (p-value = 0.0001) and CK (p-value = 0.0001). In addition, the root activities for treatments aRS + MRS and MRS were also significantly higher than aRS (p-value = 0.0011, 0.0088, respectively) and CK (both of p-values were 0.0001). The yields achieved following treatments aRS + MRS, aRS, and MRS were all significantly higher than CK (p-value = 0.0001), and the treatment of aRS + MRS had the highest yield of 28611.67 kg ha^−1^ in 2017 and 31667.33 kg ha^−1^ in 2018. Two-way ANOVA showed that the differences of plant height, root activity and yield among different treatments (all of p-values were 0.0001) and years (p-value = 0.0038, 0.0373, 0.0003, respectively) were all reaching to significant level.

**Table 3:** Biological characteristics of tomato plant after different treatments.

Treatment	Experiment time (year)	Plant height (cm)	Stem diameter (cm)	Root activity (µg g^−1^ h^−1^)	Yield (kg ha^−1^)
CK	2017	32.27 ± 1.70 c	0.53 ± 0.05 c	23.12 ± 0.66 d	6218.67 ± 621.67 e
aRS		33.33 ± 3.38 c	0.58 ± 0.00 c	26.88 ± 1.43 c	21765.67 ± 822.51 c
MRS		35.33 ± 1.29 bc	0.74 ± 0.11 b	30.04 ± 1.66 b	15236.33 ± 822.58 d
aRS + MRS		40.40 ± 1.22 a	0.84 ± 0.03 a	31.10 ± 1.15 b	28611.67 ± 619.18 b
CK	2018	34.59 ± 1.39 bc	0.53 ± 0.02 c	20.12 ± 1.81 e	5618.33 ± 518.76 e
aRS		36.49 ± 0.86 b	0.58 ± 0.02 c	26.15 ± 0.77 c	21677.67 ± 535.72 c
MRS		39.93 ± 1.21 a	0.82 ± 0.04 ab	34.20 ± 1.70 a	20575.33 ± 158.68 c
aRS + MRS		40.27 ± 2.11 a	0.83 ± 0.02 a	35.50 ± 0.43 a	31667.33 ± 582.78 a

Data are means ± standard error (n = 3); Values within a row followed by the same letter are not significantly different at P ≤ 0.05. Plant height, stem diameter and root activity were measured at vegetative stage.

### Physicochemical properties of tomato rhizosphere soils after the different treatments

The soil chemical parameters were influenced by the different treatments (Table 4). Compared with CK, the application of MRS alone or in combination with aRS significantly increased the soil pH, by changing it from acidic (CK, pH 4.5 in 2017 and 4.4 in 2018) to nearly neutral (pH 6.57 in 2017 and 7.07 in 2018 for the treatment of MRS + aRS). The MRS and aRS + MRS treatments also significantly increased the soil organic carbon (SOC), the soil total nitrogen (TN), the total phosphorus (TP) and the total potassium (TK) contents compared with CK (all of p-values were 0.0001). For example, the SOC contents aRS + MRS treatment were 63.49 g kg^−1^ in 2017 and 82.90 g kg^−1^ in 2018, which were approximately 3-fold or 4-fold higher than that of CK (21.47 g kg^−1^ in 2017 and 19.09 g kg^−1^ in 2018). Moreover, the SOC, TN, TP, TK and exchangeable calcium contents of MRS and aRS + MRS treatments in 2018 were significantly higher than those in 2017 (all of p-values were 0.0001).

**Table 4:** Properties of tomato rhizosphere soils after the different treatments.

Treatments	Experiment time (year)	Items					
		pH	#SOC (g kg^−1^)	TN (g kg^−1^)	TP (g kg^−1^)	TK (g kg^−1^)	Exchangeable calcium (cmol kg^−1^)
CK	2017	4.50 ± 0.10 e	21.47 ± 1.30 e	1.63 ± 0.02 d	1.14 ± 0.05 d	13.25 ± 0.16 c	6.20 ± 0.02 d
aRS		4.87 ± 0.06 d	27.55 ± 0.68 d	1.73 ± 0.02 c	1.89 ± 0.01 c	15.34 ± 0.09 b	6.13 ± 0.03 d
MRS		6.17 ± 0.06 c	54.69 ± 2.13 b	1.83 ± 0.03 b	2.21 ± 0.02 b	15.90 ± 0.07 b	6.63 ± 0.06 cd
aRS + MRS		6.57 ± 0.06 b	63.49 ± 2.30 c	1.81 ± 0.01 b	2.14 ± 0.06 b	15.93 ± 0.15 b	7.47 ± 0.05 c
CK	2018	4.40 ± 0.10 e	19.09 ± 1.04 e	1.61 ± 0.01 d	1.03 ± 0.12 d	11.85 ± 0.14 d	6.28 ± 0.14 d
aRS		4.77 ± 0.15 d	25.98 ± 2.97 d	1.82 ± 0.03 b	1.92 ± 0.04 c	16.06 ± 0.45 b	6.08 ± 0.07 d
MRS		7.00 ± 0.20 a	77.61 ± 4.12 a	1.96 ± 0.02 a	3.00 ± 0.05 a	18.30 ± 0.21 a	14.57 ± 1.05 b
aRS + MRS		7.07 ± 0.23 a	82.90 ± 2.72 a	1.97 ± 0.01 a	3.02 ± 0.04 a	18.40 ± 0.22 a	16.67 ± 1.40 a

Data are means ± standard error (n = 3); Values within a row followed by the same letter are not significantly different at P ≤ 0.05. #Soil organic carbon (SOC), total nitrogen (TN), total phosphorus (TP), total potassium (TK) and Exchangeable calcium.

### Enzymatic activity in the tomato rhizosphere soils after the different treatments

**Figure 3:**
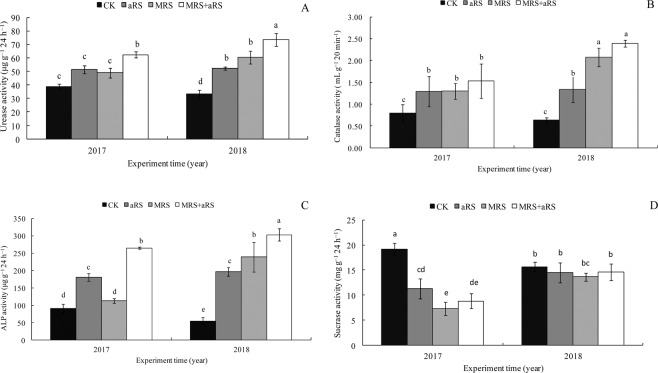
Effect of different treatments on the soil enzymatic activity. A urase, B catalase, C alkaline phosphatase, and D sucrase. aRS, avirulent *Ralstonia solanacearum* strain FJAT-1458 inoculation alone; MRS, soil amendment with microbial restoration substrate alone; aRS + MRS, combination of aRS and MRS; CK, non-(aRS or MRS) was used as control. Bars represent the means of three replicates for each treatment with error bars denoting the SE. Different lower-case letters above the bars indicate statistically significantly differences according to LSD test.

The urease, catalase and alkaline phosphatase (ALP) activities for aRS, MRS and aRS + MRS treatments were all significantly higher than CK both in 2017 and 2018 experiments (P < 0.05) (Fig. 3A–C). The aRS + MRS treatment showed maximum urease, catalase and ALP activities in the two consecutive years (Fig. 3A–C). Compared to CK, the application of aRS or MRS alone or in combination significantly decreased sucrase activity by 41.25%, 62.18% and 54.06%, respectively in 2017 (all of p-values were 0.0001) (Fig. 3D).

### Key soil factors taking part in BW control

A correlation analysis was conducted between BW disease incidence and soil properties in order to obtain the key soil factors involved in the process of BW control in tomato. The results showed that disease incidence was negatively correlated with soil pH and with SOC, TN, TP, TK and the exchangeable calcium contents, with correlation coefficients of −0.54, −0.62, −0.42, −0.71, −0.84 and −012, respectively (Table 5), which indicated that high soil pH and high SOC, TN, TP, TK and exchangeable calcium contents are beneficial for plant health.

**Table 5:** Pearson correlation analysis between disease incidence and soil properties.

	pH	SOC	TN	TP	TK	Exchangeable calcium
r	−0.54	−0.62	−0.42	−0.71	−0.84	−0.12

r indicates correlation coefficient; SOC, Soil organic carbon; TN, total nitrogen; TP, total phosphorus; TK, total potassium.

## Discussion

Tomato BW results in huge economic losses each year in many tropical and subtropical areas^24^. Various approaches including bactericide application^40^, crop rotation^7^, anaerobic soil disinfestation^41,42^, biological agents^43^ have been attempted to arrest the disease so far. The use of chemical bactericide is associated toxic residues and environmental pollution. Biological control is a non-hazardous alternative for integrated pest management. However, it is difficult to achieve satisfactory disease control using biological agent alone. Many studies report that the combination of biocontrol agents and soil amendment enhances the control efficiency of soilborne disease compared with either treatment alone^44–46^. Our study revealed that the application of aRS + MRS in combination had significantly higher control efficiency than the application of aRS and MRS alone. This may be attributed to the additive effects of aRS and MRS in promoting each other establishment in the rhizosphere, because aRS and MRS act through different mechanisms to combat disease. Similar results were reported by Singh *et al*.^44^, showing that combining biocontrol agents and soil amendments improved the control of *Macrophomina phaseolina-* and *Fusarium-* induced diseases in legume and spice crops.

The ability of biocontrol bacteria to efficiently colonize the rhizosphere is a key factor in their successful improvement of plant health and suppression of plant pathogens^47,48^. Kempe and Sequeira (1983) had reported that a high density of avirulent *R*. *solanacearum* within plant tissues prevented or delayed pathogen entry into the host^13^. In this study, the number of avirulent *R*. *solanacearum* in the tomato rhizosphere soil was significantly higher for the combined application of aRS and MRS than the application of aRS alone, which was consistent with a lower DSI value for aRS + MRS than for aRS. This could be because MRS improved the soil physicochemical characteristics, such as soil pH and nutrients’ availability, which increased the colonization ability of aRS.

Decreasing virulent *R*. *solanacearum* population in the soil is important for soil suppression of BW^9^. Soil amendment with compost or manure^49,50^ or pig slurry^51^ reduced the populations of soilborne pathogens, including *R*. *solanacearum*. Ghosh *et al*.^52^ reported that the integration of organic and inorganic soil amendments indeed suppressed the growth of *R*. *solanacearum*^52^. In this study, the applications of aRS and MRS alone or in combination directly suppressed the growth of *R*. *solanacearum* in the tomato rhizosphere, especially for the combination using aRS and MRS. Similarly, Khan *et al*.^53^ reported that the population of the *R*. *solanacearum* virulent strain was reduced in rhizosphere after the application of *R*. *solanacearum* avirulent strain^53^. The avirulent strain exhibited antagonism toward BW pathogen under culture, glasshouse and field conditions^12–14,54^.

Compared to CK, the SOC, TN, TP, TK, and exchangeable calcium contents and the soil pH were significantly increased with increasing application of MRS, SOC and TK contents increased and exchangeable calcium concentration decreased with increasing application of aRS. A Pearson correlation analysis indicated that soil pH, and the SOC, TN, TP, TK and exchageable calcium contents were negatively related to BW disease incidence, which was in agreement with previous reports^9,55,56^. For example, the results of Liu *et al*. (2015) revealed that the SOC, TN, TP and TK contents played a key role in preventing the tomato plant from being infected by the wilt pathogen^9^. Yamazaki^57^ reported that an increase in the Ca concentration in tomato plants decreased the incidence of BW as well as *R*. *solanacearum* population^57^. Soil acidification is closely related to BW outbreak. The average soil pH in fields infected by BW was much lower than that in non-disease fields^58^. Thus, it is especially important for controlling BW by improving soil pH^59^.

The mechanism of action of nonpesticide chemicals to suppress BW is considered to involve either inducing systemic resistance or antibacterial activity^2^. Li and Dong (2013) found that the application of a commercial organic fertilizer, combined with rock dust soil amendment, increased the activities of alkaline phosphatase, urease, catalase and sucrase to a greater extent in the soil^60^. In the present study, the application of aRS and MRS alone or in combination significantly enhanced the activities of soil urease, catalase and ALP enzymes. This result is in agreement with a previous study^61^, suggesting that some plant defense enzyme activities were induced by the biocontrol agent.

In this study, we found that continuously use MRS resulted in a better crop yield and BW control. The control efficiencies, root activities, yields and soil properties from treatments of MRS and aRS + MRS in 2018 were all significantly higher than those in 2017. This indicates there are cumulative effects of MRS and aRS + MRS in the experiment of 2018. Moreover, the combined use of aRS and MRS reaching to a synergistic control effect, for its observed control efficiency higher than the expected control efficiency. Thus, the combination of MRS and aRS represents a promising new tool to control BW.

## Conclusions

The combination of MRS and aRS could significantly suppress virulent *R*. *solanacearum* survival, reduce tomato bacterial wilt disease severity, and increase root activities and tomato yield. The outcomes might mainly attributed to the “MRS + aRS” being able to change soil pH from acidic to nearly neutral, improve soil nutrients, and enhance some soil enzyme activities. In conclusion, the combined application of MRS and aRS is beneficial to soil nutrient cycling and plant health, indicating a promising new tool to control tomato bacterial wilt.
